# *Memo1* gene expression in kidney and bone is unaffected by dietary mineral load and calciotropic hormones

**DOI:** 10.14814/phy2.14410

**Published:** 2020-04-14

**Authors:** Matthias B. Moor, Olivier Bonny

**Affiliations:** ^1^ Department of Biomedical Sciences University of Lausanne Lausanne Switzerland; ^2^ The National Centre of Competence in Research (NCCR) "Kidney.CH ‐ Kidney Control of Homeostasis" Switzerland Zürich Switzerland; ^3^ Service of Nephrology Department of Medicine Lausanne University Hospital Lausanne Switzerland; ^4^ Present address: Department of Nephrology and Hypertension Bern University Hospital Bern Switzerland

**Keywords:** calcium, FGF23, Memo1, phosphate, vitamin D_3_

## Abstract

Mediator of cell motility 1 (MEMO1) is a ubiquitously expressed modulator of cellular responses to growth factors including FGF23 signaling, and *Memo1*‐deficient mice share some phenotypic traits with *Fgf23*‐ or *Klotho*‐deficient mouse models. Here, we tested whether *Memo1* gene expression is regulated by calciotropic hormones or by changing the dietary mineral load. MLO‐Y4 osteocyte‐like cells were cultured and treated with 1,25(OH)_2_‐vitamin D_3_. Wild‐type C57BL/6N mice underwent treatments with 1,25(OH)_2_‐vitamin D_3_, parathyroid hormone, 17β‐estradiol or vehicle. Other cohorts of C57BL/6N mice were fed diets varying in calcium or phosphate content. Expression of *Memo1* and control genes was assessed by qPCR. 1,25(OH)_2_‐vitamin D_3_ caused an acute decrease in *Memo1* transcript levels in vitro, but not in vivo. None of the hormones tested had an influence on *Memo1* transcripts, whereas the assessed control genes reacted the expected way. Dietary interventions with calcium and phosphate did not affect *Memo1* transcripts but altered the chosen control genes’ expression. We observed that *Memo1* was not regulated by calciotropic hormones or change in mineral load, suggesting major differences between the regulation and physiological roles of *Klotho*, *Fgf23*, and *Memo1*.

## Graphical abstract

MEMO1, a novel regulator of mineral homeostasis, is indifferent to dietary calcium or phosphate load and calciotropic hormones such as 1,25(OH)_2_‐vitamin D_3_, parathyroid hormone, and 17β‐estradiol.

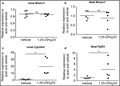

## 1 INTRODUCTION

*Memo1* is an evolutionary conserved protein in all kingdoms of life that has shown intracellular expression in cytoplasma and nucleus (Haenzi et al., 2014; Moor, Haenzi, et al., 2018; Schlatter et al., 2012). A conditional and inducible knockout mouse model with postnatal deletion of exon 2 of the *Memo1* gene has resulted in a syndrome of aging and premature death with traits such as elevated calcemia, elevated FGF23 and 1,25(OH)_2_‐vitamin D_3_, bone disease, lung emphysema, atrophy of subcutaneous fat, insulin hypersensitivity, and renal insufficiency (Haenzi et al., 2014; Moor, Ramakrishnan, et al., 2018). This phenotype significantly overlaps with phenotypes of mouse models deficient in KLOTHO (Kuro‐o et al., 1997) or FGF23 (Shimada et al., 2004), two proteins which have tremendously reshaped our understanding of the regulation of calcium and phosphate metabolism by the kidney and bone and to a lesser extent also the intestine (Hu, Shiizaki, Kuro‐O, & Moe, 2013; Moor & Bonny, 2016).

In addition, evidence from cell culture experiments investigating co‐localization and phosphorylation status of adaptor proteins suggested that mediator of cell motility 1 (MEMO1) protein participates in and modulates a signaling cascade involving FGF23 and the FGFR (Haenzi et al., 2014).

Serum analyses of *Klotho* and *Fgf23*‐deficient mice showed excessive 1,25(OH)_2_‐vitamin D_3_ levels, a finding that has been variably found in *Memo1*‐deficient mice depending on the genetic background (Haenzi et al., 2014; Moor, Ramakrishnan, et al., 2018). The promoters of *Fgf23* and *Klotho* both contain vitamin D response elements (VDRE) (Forster et al., 2011; Orfanidou, Malizos, Varitimidis, & Tsezou, 2012). FGF23 secretion is increased by parathyroid hormone (PTH) (Lavi‐Moshayoff, Wasserman, Meir, Silver, & Naveh‐Many, 2010) and 17β‐estradiol (Carrillo‐Lopez et al., 2009). Regarding the regulation of *Memo1*, a transcriptomic analysis of rat pineal glands has detected increased *Memo1* transcripts upon synthetic estrogen treatment compared to controls (Deffenbacher & Shull, 2006), highlighting a potential endocrine regulation of the transcription of the *Memo1* gene. Here, we, therefore, tested the hypothesis that *Memo1* expression can be regulated by minerals or calcitropic stimuli.

## 2 METHODS

### 2.1 Cell culture

The mouse long bone osteocyte‐Y4 cell line (MLO‐Y4) was kindly provided by Lynda Bonewald (Kato, Windle, Koop, Mundy, & Bonewald, 1997). MLO‐Y4 cells were maintained in culture in minimal essential medium alpha‐modified, alpha‐MEM (Gibco by Life Technologies), containing 2.5% heat‐inactivated calf serum (Sigma), 2.5% heat‐inactivated fetal bovine serum (Sigma), and 1% penicillin/streptomycin (Invitrogen by Life Technologies). Serum heat inactivation was carried out in water bath at 56°C for 30 min. Cells were cultured on rat‐tail type I collagen (Invitrogen by Life Technologies). For vitamin D stimulation, cells were kept in serum‐free medium supplemented with 10 nM 1,25(OH)_2_‐vitamin D_3_ (Sigma) or ethanol vehicle for 24 hr.

### 2.2 Animal experiments

C57BL/6N mice were obtained from Janvier. Mice were held in a conventional animal facility with up to six animals per cage and they were fed a standard laboratory chow (Kliba Nafnag TS3242; calcium 1%, phosphorus 0.65%, magnesium 0.23%, vitamin D 1,600 IU/kg, vitamin A 27,000 IU/kg, vitamin E 150 mg/kg, protein 18.8%, crude fat 5.6%, crude fiber 3.5%, lysine 1.1%; KLIBA) unless specified otherwise and were kept on 12/12 (experimental) or 14/10 (breeding) light–dark cycles. All animal experimental protocols were approved by the State Veterinary Service of the Canton de Vaud, Switzerland. For all mouse studies, sample sizes were considered based on previous results in our laboratory.

### 2.3 Dietary interventions

For studies of mineral metabolism, specifically designed diets and hormones were used. Calcium diet challenge experiments were carried with five male C57BL/6N mice per condition in their home cage, all aged 12 weeks. Mice were randomly allocated to be fed modifications of KLIBA 2222 diet containing either 0.17% (low calcium diet), 0.82% (normal calcium diet) or 1.69% (high calcium diet) (KLIBA, 2222) over 7 days. All calcium diets contained phosphorus 0.8%, vitamin A 4,000 IU/kg, vitamin D 1,000 IU/kg, vitamin E 100 mg/kg, protein 18%, and crude fat 7%, lysine 14 g/kg (Kliba, 2222). For a phosphate diet challenge, three groups of five male C57BL/6N mice all aged 13–14 weeks were kept in metabolic cages and randomly allocated to be fed diets containing low (0.2%), intermediate (0.8%), or high (1.5%) phosphate content over 7 days. The phosphate diets were modifications of KLIBA 2222 diet (calcium 1.2%, vitamin A 4,000 IU/kg, vitamin D 1,000 IU/kg, vitamin E 100 mg/kg, protein 18%, crude fat 7%, and lysine 14 g/kg).

### 2.4 Hormone injections

All hormone injections were performed in the home cage of the mice after random treatment allocation of individual ear‐marked mice. For 1,25(OH)_2_‐vitamin D_3_ treatment, male C57BL/6N mice aged 13–15 weeks were subcutaneously injected with 2 μg/kg body weight 1,25(OH)_2_‐vitamin D_3_ (Sigma D1530) in ethanol 1% in NaCl 0.9%. The dose and application route was the same as previously used in our laboratory. Control mice were injected with 1% (v/v) ethanol in NaCl 0.9%. Mice were sacrificed 6 hr after injection.

For PTH treatment, male C57BL/6N mice aged 12–13 weeks were subcutaneously injected with 80 μg/kg body weight human PTH fragment 1–34 (hPTH1‐34) (Sigma P3796) in NaCl 0.9% or NaCl 0.9% alone as vehicle and sacrificed 2 hr after injection. The dose and application route used was determined from the literature (Kramer, Loots, Studer, Keller, & Kneissel, 2010).

For estradiol treatment, male C57BL/6N mice aged 16 weeks received one daily subcutaneous injection of 15 μg 17β‐Estradiol (Sigma E8875) in ethanol 0.1% (v/v) in NaCl 0.9% for five consecutive days and were sacrificed 4 hr after the last injection. The dose per body weight and the drug application route was derived from the literature, as shown Van Abel et al. (2002) to induce *Trpv5* expression. Control mice were subcutaneously injected with 0.1% ethanol in NaCl 0.9% for 5 days.

### 2.5 Mouse dissection

For euthanasia, mice were intraperitoneally injected with 0.1 mg/gBW of ketamine (Ketanarkon 100 Vet., Streuli) and 0.02 mg/gBW of xylazine (Rompun, Bayer), followed by terminal exsanguination by orbital puncture under full anesthesia and/or by cervical dislocation. Organs were collected, kidneys were cut in half, and organs were snap frozen in liquid nitrogen immediately, followed by storage at −80°C until further use.

### 2.6 RNA extraction

RNA was extracted using TRI reagent (Applied Biosystems by Life Technologies) according to manufacturer's instructions. RNA pellets were dried and dissolved in RNase‐free H_2_O. RNA concentration was measured photometrically using NanoDrop (NanoDrop 2000, Thermo Fisher Scientific). RNA A260/A280 ratio was assessed and each RNA sample was visualized on a 1% agarose gel.

RNA was reverse transcribed to cDNA using the PrimeScript RT reaction kit (Takara Bio Inc). RNA input quantities per sample were 1–2 μg for bone, 500 ng for kidney or 1 μg of MLO‐Y4 RNA. The resulting cDNA mix was diluted 2–12x depending on tissue type.

### 2.7 qPCR

For quantitative gene transcript expression analysis, 2 μL of cDNA was used for SYBR Green qPCR (Applied Biosystems by Life Technologies) on a 7500 Fast machine (Applied Biosystems). Samples were run in triplicate in 20 μL total volume for each gene, and actin or GAPDH was used for normalization. Melting curves were obtained for every run. Program settings were: 95°C during 20 s, 40 cycles (95°C 3 s, 60°C 30 s), and for melting curve stage: 95°C 15 s, 60°C 1 min, rising at 1% ramp speed to 95°C (15 s), and 60°C 15 s. Data were analyzed using the delta‐delta CT method. Primers were ordered from Microsynth (Switzerland) and sequences are shown in Table 1. All amplified products were visualized on agarose gels.

**Table 1:** Primers used for qPCR

Oligonucleotide	5'‐sequence‐3'
Memo1 forward	GCTGCCCATGCTTACAAACAA
Memo1 reverse	AGAGTGCACATCGAGACAGG
Rankl forward	GTCTGTAGGTACGCTTCCCG
Rankl reverse	CATTTGCACACCTCACCATCAAT
Bglap forward	CCGCCTACAAACGCATCTATG
Bglap reverse	GCTGCTGTGACATCCATACTTG
Phex forward	GTGCATCTACCAACCAGATACG
Phex reverse	TCTGTTCCCCAAAAGAAAGG
Slc34a3 forward	CCTACCCCCTCTTCTTGGGT
Slc34a3 reverse	AGAGCAACCTGAACTGCGAA
F3 forward	ACCTGGGCCTATGAAGCAAA
F3 reverse	GTTGGTCTCCGTCTCCATGAA
Cyp27b1 forward	ATGTTTGCCTTTGCCCAGA
Cyp27b1 reverse	GACGGCATATCCTCCTCAGG,
Cyp24a1 forward	GAAGATGTGAGGAATATGCCCTATTT
Cyp24a1 reverse	CCGAGTTGTGAATGGCACACT
beta‐actin forward	GTCCACCTTCCAGCAGATGT
beta‐actin reverse	AGTCCGCCTAGAAGCACTTGC

### 2.8 Data analysis

Human *Memo1* promoter sequences were analyzed in silico using the UCSC Genome Browser and Serial Cloner 2.6.1.

Data from experiments with two independent groups were analyzed by *t* test or Mann–Whitney *U* test. For comparison of three groups, Kruskal–Wallis test was used with Bonferroni's Multiple Comparison posttest. All statistical analyses were conducted using GraphPad PRISM 5.03. Two‐sided *p* < .05 were considered significant.

## 3 RESULTS

*Memo1*‐deficient mice resemble by some traits *Klotho* mutant and *FGF23* KO mice (Haenzi et al., 2014), and the promoters of *Klotho* and *FGF23* harbor regulatory sequences that can be bound by vitamin D receptors (Forster et al., 2011; Orfanidou et al., 2012). For these reasons, we determined the regulation of *Memo1* gene expression by minerals and calciotropic hormones. We have previously shown that MLO‐Y4 osteocyte‐like cell line expressed *Memo1* transcripts and protein (Moor, Ramakrishnan, et al., 2018).

### 3.1 *Memo1* is diminished by 1,25(OH)_2_‐vitamin D_3_ in vitro but not in vivo

**Figure 1:**
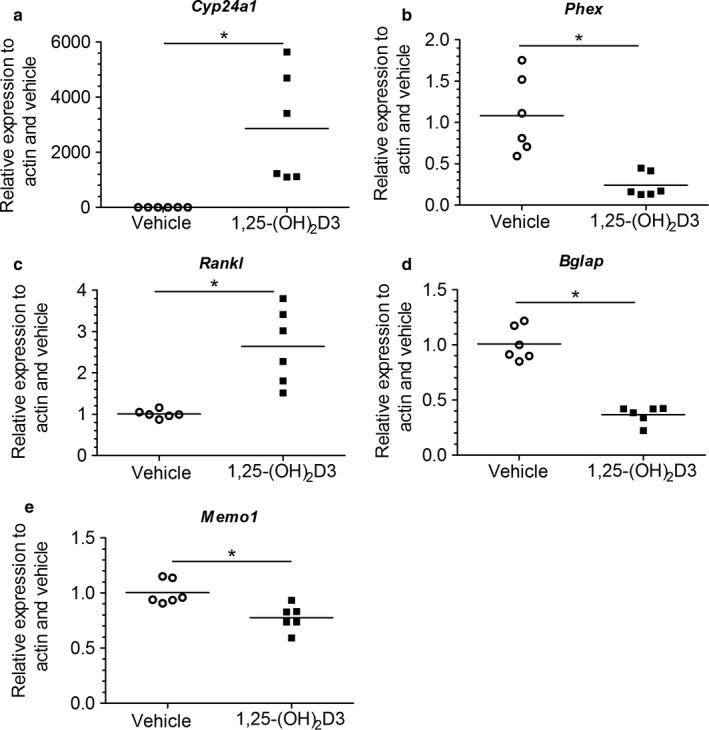
Transcriptional responses to 1,25(OH)_2_‐vitamin D_3_ in MLO‐Y4 osteocyte‐like cells. 1,25(OH)_2_‐vitamin D_3_ treatment significantly increased *Cyp24a1* transcripts (a), decreased *Phex* transcripts (b), increased *Rankl* transcripts (c), decreased *Bglap* (osteocalcin) transcripts (d), and decreased *Memo1* (e) transcripts in MLO‐Y4 cells. *n* = 6 per condition, *p < .05 (*t* test)

An in silico promoter analysis of the human *Memo1* gene revealed a conserved CpG island (Gardiner‐Garden & Frommer, 1987) sequence in the 1,000 bases in 5’ direction of transcription start site of *Memo1* that was considered as the putative promoter sequence (Figure S1). In a screen for published VDREs, we identified two incidences of a negative VDRE with the sequence 5′‐GCTTTCC‐3′ (Towers, Staeva, & Freedman, 1999). Five VDRE sequences reported elsewhere (Calle, Maestro, & Garcia‐Arencibia, 2008; McGaffin & Chrysogelos, 2005; Roff & Wilson, 2008) were undetectable on either sense or antisense strand. Therefore, we proceeded to experimentally investigate the effects of stimulation with 10 nM 1,25(OH)_2_‐vitamin D_3_ on gene expression in MLO‐Y4 cells (Figure 1). Known vitamin D‐dependent transcripts were first assessed. Transcripts of *Cyp24a1* encoding a vitamin D inactivating hydroxlyase (Figure 1a) and of osteoclast regulator *Rankl* (Figure 1c) were increased upon 1,25(OH)_2_‐vitamin D_3_ treatment, whereas transcripts of FGF23 regulator *Phex* and expression of bone‐derived hormone *osteocalcin*/*bone gamma‐carboxyglutamate* (*Bglap*) were diminished (Figure 1b,d). *Memo1* transcripts were reduced by 20% upon 1,25(OH)_2_‐vitamin D_3_ treatment (Figure 1e).

**Figure 2:**
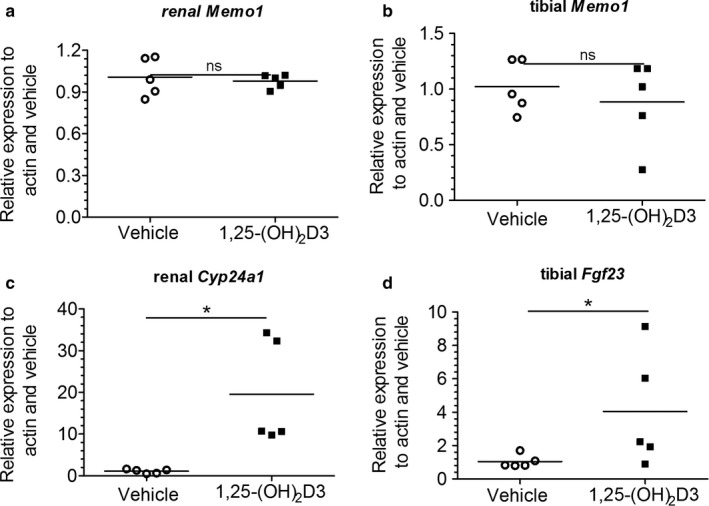
*Memo1* transcripts were not changed by 1,25(OH)_2_‐vitamin D_3_ treatment in wild‐type mice. *Memo1* transcripts were assessed in kidney (a) and tibia (b) and were indifferent 6 hr after 1,25(OH)_2_‐vitamin D_3_ injection, whereas renal *Cyp24a1* transcripts were over 10‐fold increased in comparison to vehicle control (c). *Fgf23* gene expression in tibia was increased by 1,25(OH)_2_‐vitamin D_3_ compared to vehicle (d). *n* = 5 per condition, *p < .05 (Mann–Whitney *U* test)

Next, we intraperitoneally injected 1,25(OH)_2_‐vitamin D_3_ in mice. Six hours post‐injection we harvested the kidney and tibia of these animals and investigated the mRNA levels of *Memo1*. *Memo1* RNA levels remained unchanged in the kidney (Figure 2a) and tibia (Figure 2b) compared to control animals injected with vehicle only. As experimental controls, we chose *Cyp24a1* and *Fgf23*. Renal transcripts of *Cyp24a1*, the gene encoding the vitamin D inactivating enzyme cytochrome P450 24a1, were upregulated by 1,25(OH)_2_‐vitamin D_3_ compared to vehicle (Figure 2c). In addition, expression of *Fgf23* in the tibia was increased by 1,25(OH)_2_‐vitamin D_3_ (Figure 2d).

### 3.2 *Memo1* is not regulated by dietary calcium

**Figure 3:**
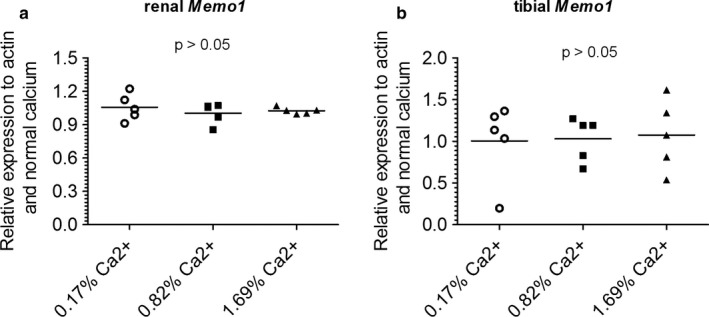
*Memo1* transcript levels were not influenced by varying dietary calcium contents. *Memo1* transcripts in kidney (a) and tibia (b) were not significantly affected by different dietary calcium intakes; *n* = 5 per diet (Kruskal–Wallis tests). Calcium‐sensing receptor increased 2.5‐fold in bone of mice on 0.17% calcium diet published in (O'Seaghdha et al., 2013) using the same samples serves as an experimental control

Next, we determined the effect of varying dietary calcium content for 7 days on *Memo1* gene expression. RNA was obtained from a previous experiment performed in our lab (366). In the kidney (Figure 3a) and in the tibia (Figure 3b) of these mice exposed to three different calcium‐containing diets (0.17%, 0.82%, and 1.69%), no change in *Memo1* gene expression was observed. A 2.5‐fold increase in gene expression of *Casr* encoding the calcium‐sensing receptor in the bone upon dietary calcium restriction serves as an experimental control for the dietary intervention and was reported for the samples we used in (O'Seaghdha et al., 2013).

### 3.3 *Memo1* is not regulated by dietary phosphate

**Figure 4:**
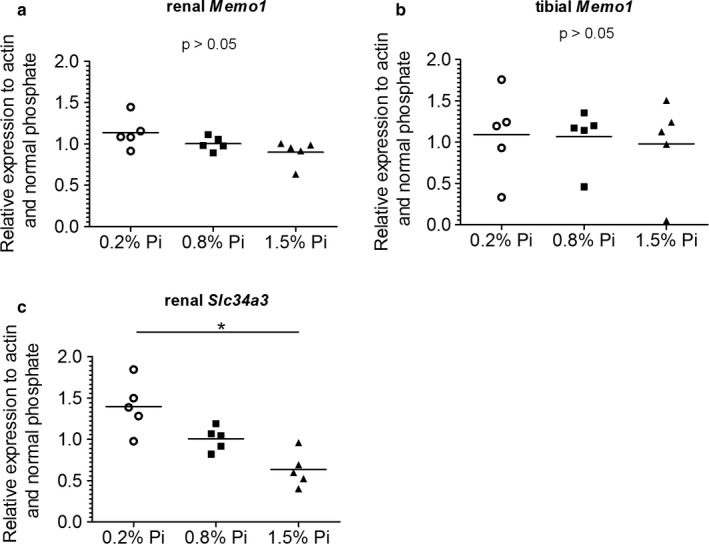
*Memo1* transcript levels were not affected by varying dietary phosphate contents. *Memo1* transcripts abundance in kidney (a) and in tibia (b) were not significantly changed by dietary phosphate contents. Neither bone *Memo1* (b). Renal *Slc34a3* transcripts were used as an experimental control gene and were affected by dietary phosphate content (c). *N* = 5 per condition; *p < .05 (Kruskal–Wallis test with Dunn's multiple comparisons correction)

We investigated the influence of different systemic phosphate loads on *Memo1* expression. We showed that different dietary phosphate contents (0.2%, 0.8%, 1.5%) did not significantly affect *Memo1* transcript levels in kidney (Figure 4a) or in the tibia (Figure 4b). As an experimental control gene we used renal transcripts of *Slc34a3* encoding sodium‐dependent phosphate transporter type 2c (NaPi2c). Renal *Slc34a3* was increased, as expected, under low phosphate and decreased under high phosphate diets (Figure 4c).

### 3.4 *Memo1* unchanged by PTH

**Figure 5:**
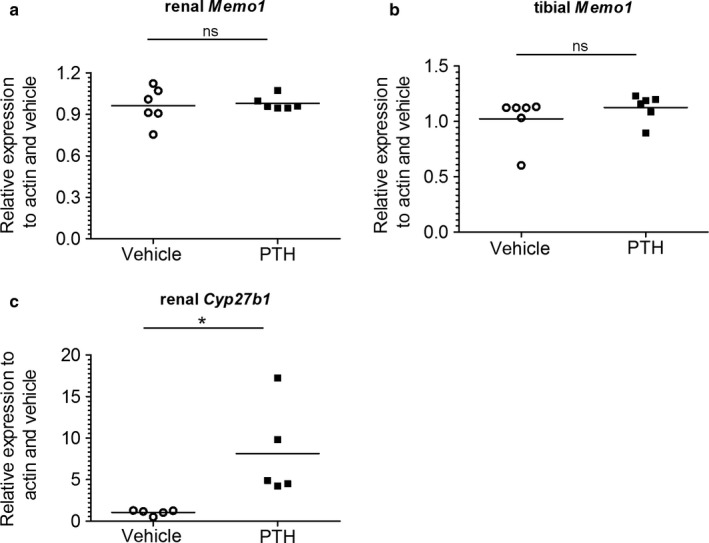
*Memo1* gene expression remained unchanged upon PTH treatment in wild‐type mice. Human PTH1‐34 or NaCl 0.9% vehicle was injected 2 hr prior to dissection and transcripts of *Memo1* in kidney (a) and tibia (b) were unchanged between experimental conditions. Transcripts of *Cyp27b1* (c) were increased by PTH1‐34. *n* = 6 per condition; ns, not significant; *p < .05 (Mann–Whitney *U* test). PTH, parathyroid hormone

To determine the effect of PTH on *Memo1*, human PTH fragments 1–34 were subcutaneously injected to wild‐type mice, and the animals were euthanized after 2 hr. *Memo1* gene expression in kidney (Figure 5a) or in tibia (Figure 5b) remained unchanged upon PTH treatment. Transcripts of *Cyp27b1*, the gene coding for the renal vitamin D activating enzyme cytochrome P450 27b1 were increased upon PTH compared to NaCl 0.9%‐treated controls (Figure 5c).

### 3.5 *Memo1* unchanged by estradiol

**Figure 6:**
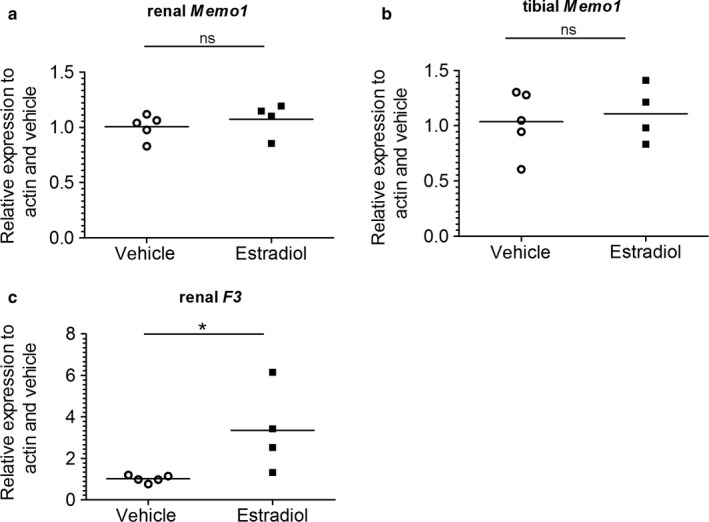
*Memo1* transcripts remained unchanged upon 17β‐estradiol treatment. *Memo1* transcripts assessed by qPCR in kidney (a) and tibia (b) were unchanged after five daily subcutaneous injections of 17β‐estradiol compared to vehicle. Renal gene expression of tissue factor *F3* was increased by 17β‐estradiol (c). *n* = 4 estradiol and *n* = 5 control condition. ns, not significant; *p < .05 (Mann–Whitney *U* test)

As sex hormones exert effects on both renal calcium transport proteins (van Abel et al., 2002) and FGF23 in the bone (Carrillo‐Lopez et al., 2009), we tested if *Memo1* is a target gene induced by estradiol. We subcutaneously injected 17β‐estradiol once daily over 5 days. This induced the expression of the control gene *F3* encoding coagulation factor III (Figure 6c), but gene expression of *Memo1* in the kidney (Figure 6a) and in the bone (Figure 6b) both remained unchanged compared to mice injected with vehicle.

To summarize, we found a small but significant decrease in *Memo1* expression upon 1,25(OH)_2_‐vitamin D_3_ exposure in vitro, but we failed to detect any major regulation of *Memo1* transcript abundance upon mineral load or calciotropic hormone treatment in vivo.

## 4 DISCUSSION

MEMO1 is expressed in the kidney where it plays an intrarenal role in the regulation of calcium transporters (Moor, Haenzi, et al., 2018). In the bone, MEMO1 is expressed in all cell types (Moor, Ramakrishnan, et al., 2018), but its precise bone‐specific function remains elusive.

Here we tested the hypothesis whether *Memo1* is regulated by key players in mineral homeostasis such as calciotropic hormones or dietary calcium or phosphate. As a readout, we chose *Memo1* gene expression in an osteocyte‐like cell line, and in bone and kidney tissues. For each intervention, an experimental control gene was assessed and revealed effects similar as shown before by others.

We observed that *Memo1* gene expression was diminished in the osteocyte‐like cells upon 1,25(OH)_2_‐vitamin D_3_ treatment. However, in bone and tissues, we failed to detect any effect on *Memo1* by all interventions that we studied. This shows a major difference between *Memo1* and the most studied contributors to mineral homeostasis. As examples in the kidney, Type II sodium‐dependent phosphate cotransporters are regulated by dietary phosphate supply (Bourgeois et al., 2013) and gene expression of *Trpv5* encoding a renal calcium transport protein is controlled by 1,25(OH)_2_‐vitamin D_3_ (Hoenderop et al., 2001). As examples in the bone, *Fgf23* expression is stimulated by 1,25(OH)_2_‐vitamin D_3_ (Liu et al., 2006) or PTH (Kawata et al., 2007), while dietary phosphate restriction or renal phosphate‐wasting disorders reduce *Fgf23* expression (Ansermet et al., 2017; Schlingmann et al., 2016; Vervloet et al., 2011). Even intravenous calcium loading in rats increased *Fgf23* expression in bone and hormone concentrations in the serum (Shikida et al., 2018).

This study contains some limitations: The current interventions were confined to the analysis of gene expression, but we did not directly assess *Memo1* promoter activity using a reporter construct. Such an approach would more sensitively discriminate and would allow validating putative response elements in the *Memo1* promoter. In addition, our in silico analysis of the presumed promoter sequence did not allow base mismatches compared to known response elements. However, we argue that a physiologically relevant the regulation of *Memo1* gene expression, if present, should have been visible using the experimental approaches that were undertaken.

Further, we have assessed a single but physiologically reasonable time point, and only a narrow selection of tissues and cells. In addition to bone and kidney, the intestine would be another major turnover place for minerals. *Memo1* expression and potential regulation in healthy intestine have not been investigated so far. In colorectal cancer cells *Memo1* promoter activity is increased in response to the transcription factors Aryl hydrocarbon receptor/ Aryl hydrocarbon receptor nuclear‐translocator complex, indicating some intestinal disease relevance (Bogoevska et al., 2017).

Another limitation is the fact that we investigated only mice of male sex as to simplify experimental planning, reduction of mice numbers used, and as to reproduce the hormone injection protocols in the cited references, including the estradiol injection protocol (van Abel et al., 2002). Future experiments should be conducted with both sexes independently to allow the detection of sex‐specific effects.

Finally, as Memo is a redox enzyme with incompletely understood reaction partners and substrates (MacDonald et al., 2014), an assessment of posttranslational regulation such as by the oxidative modification of MEMO1 protein, subcellular localization, or changes in its putative enzymatic activity may be helpful to investigate a regulation of Memo1.

To conclude, besides a minor effect in bone cells stimulated with 1,25(OH)_2_‐vitamin D_3_, we did not detect a major regulation of *Memo1* gene expression upon minerals and calciotropic stimuli in bone and kidney, two organs relevant for mineral homeostasis. Further studies inquiring the regulation of this and similar genes may contribute to the understanding of the regulation of mineral homeostasis in health and renal and bone diseases.

## CONFLICT OF INTEREST

The authors declare that no conflict of interest exists.

## AUTHOR CONTRIBUTIONS

OB conceived the project. MBM and OB participated in the experimental design. MBM performed the experiments. MBM and OB participated in the data analysis and interpretation. MBM wrote the manuscript. All authors critically read and commented on the manuscript and agreed to the manuscript submission.

## Supporting information



Fig S1

## Data Availability

Raw data are available from the authors on request.
