# Supplementary material for: Memo1 gene expression in kidney and bone is unaffected by dietary mineral load and calciotropic hormones
Source: Physiol Rep. 2020 Apr 14;8(7):e14410. doi: 10.14814/phy2.14410 (PMC7156332; doi:10.14814/phy2.14410)
Supplement: Supplementary file 1 — Fig S1 [file PHY2-8-e14410-s001.pdf]

```
>hg19_cpgIslandExt_CpG: 115 range=chr2:32234654-32236248 5'pad=0 3'pad=0
strand=+ repeatMasking=none
5’-CGGGGCGAGAAGACTTCCTCTCCGGCTGCGGGCGCGGGGGCTCGGGGGCA
GACCGAGAGAACTCCCTCTGGGCGAGGGCACTCCCCGGACCGCCAGCGGG
GAGGGCAGGGAAGACGGGAGAGGAAAGGAAAGGGTCCCTGGCTCGGCAAG
AGGGCGGAGGGTCGCTGCCCTGCGGCGTGGAGAAGCGGGGCTCGGCGCCG
GGCACACCGTCCTTCCCGGGGGTGGCAGCGGCCGGCCGCACGCTTCCTGC
AAGCCAGAGGCGGCGACAAGTCGGCTGGGGTCCGGGAGCACGTTCCTTCC
TCGAGCCTGGTCCTGAGGGAGGGCGCAGGCTGCCGAGGCGGCAACTCTCC
CTCTCGCCCAACTCCGGAGAGCGTGAGGCCGGCCGGCCCCGGGCTGCCGG
CGCGGGATGCCCGCCGCCCGGGTGGGGGAGGCGGCGAGGCCGGCCTCGGC
TCGCTCCCTCCCCACGCGGTCCGCGCCCGGCGGCCCGTCCGCGCCCTCTT
CCCCGCCCGCCGCCGCCGCGGGGCCGGGCCGCCGACCTCGGCCGGCCGGG
CGTGGGGCCAGGCGGCGACGGCGGCGGGCGGGCCGGCGGCCTGGGGCCCT
ACCTGAGGCTGTGTACCAGCTCCCGGCGTGACTGGCTTCTCGGCAGACCA
CTCGGTTGGACATCTTGGTGCCTGTGCCGCCTATGGTGCACGAGGATGAA
TGAGGAGGCGGCGGCGGCGGCGGCAGGAGCGGCTCCGCGAGGGGACGAGA
CACCGCGGGCCCAGCCCAGGAGGAGGCGGCAGCGGGGAGGGGATCAGCCC
GGCCCAGGAGGAGGAGGAAGAGGAGGAGGCGGCGGCCCAGGAGGAGGAGA
TGGCAGCCGGGGCGGTGGCGGCGGCAGCAACAATCACCACAAACTCCGGC
GACCGCCGGAAGATGGGGCCCACGGCGGCCTCACCGAAGCCCGGAAAGCC
CTGGCCCCGGCCCCCGGCGTCCCCGCTGCTGCCCAGCCGGGGCTGGTTCC
GCCGAGCCGCCCCCACGGCCCCCTCCCCTCCCAGCTGGCCTCCCCGAGCC
ACCGCGAGTGACCCCACCCCCGTGACTCCGCGCCCGCCCCCGCCCCCCAC
CCCCCGCGGGGCTCCCCGCACCCACCCCCACCCCCGGCAGGGCCCTCCAG
CCCGGCCGCCCGCCGCCGGCCCCGCCCCGCTCCTCCTCCCGGCCGCGCGG
CGAGGTTAACGGCGGAAACCGGACAGGCCTGACGGCATCTGGGGTCGGGC
AGCCCCGCGAGGAGGCAGTGGCGGCTGCAGCTACGCGCACACAAAAGCCC
AGGGCCGGGGCCTGAACCGGGGAAGGCAGACCCGGAGAAGAGCCAACGCT
TCCTTACTCGCCACTCTAACGGGTCCTGCCCACTGAGCATGCCCAGCACC
GCTGCCTACCCGCCGAGGAATCTCAACGTGCACGCTCCGCTCTCCGGGGT
TTAGCGGCAGCAGAGGCTGGGAGGTGGGGGCAGGGCGCGGGGAGCAGGAC
GCAGCCGCAAGGCAAGCCCCCTCCCCCAGCCAACTTCCTCAAATGGAGAG
CACCTAGGGCTCGCGGATGCGGCTTTCCACGAGGACAGTCCCGCG-3’
```

Supplemental Figure 1

Putative human *Memo1* promoter sequence.

Sequence of human chr2:32,234,654-32,236,248 is depicted containing the conserved CpG island that harbors the putative Memo1 promoter.
